# DEVELOPING THE NATIONAL STUDY OF CAREGIVING: CAREGIVING APPRAISAL MEASURE FOR CAREGIVERS OF PEOPLE WITH DEMENTIA

**DOI:** 10.1093/geroni/igae098.2741

**Published:** 2024-12-31

**Authors:** Dingyue Wang, Susan Silva, Cristina Hendrix

**Affiliations:** Duke University, Seattle, Washington, United States; Duke University, Durham, North Carolina, United States; Duke University, Durham, North Carolina, United States

## Abstract

This study aimed to develop and psychometrically validate the NSOC-Caregiving Appraisal (NSOC-CA) measure using data from the National Study of Caregiving (NSOC) IV Round 12. We also aimed to compare dementia and non-dementia caregiving experiences to determine whether this measure differentiates the caregiving experiences between the two groups. We analyzed data from 1783 adult family caregivers using principal components analysis (PCA) to identify caregiver experience domains. Varimax rotation was used to derive orthogonal factors. Hierarchical mixed-effects models, controlling for nesting effects due to some patients having multiple caregivers, were conducted to test for dementia vs non-dementia caregiver experience differences. The final 25-item PCA model was a 3-factor solution that explained 96% of the total variance. Identified domains were labeled: psychological well-being, caregiving burden, and personal impact. NSOC-CA demonstrated high reliability (Cronbach’s alpha = 0.89, for the three subscales 0.89, 0.80, 0.75 respectively). Pearson correlations of the subscale scores ranged from 0.12 to 0.46. Dementia caregivers compared to non-dementia caregivers had worse psychological well-being, greater caregiving burden, and more negative overall caregiving appraisal (all p <.0013 without covariates; and all p <.0006 with covariates) but did not differ on personal impact (p >.05). NSOC-CA is a reliable tool for assessing the caregiver experiences of those caring for patients with dementia. NCOS-CA can differentiate the caregiving experiences of those who care for family members living with and without dementia. Future research will focus on further developing the scale and assessing its psychometric properties.

